# Chemo- and bio-informatics insight into anti-cholinesterase potentials of berries and leaves of *Myrtus communis* L., Myrtaceae: an in vitro/in silico study

**DOI:** 10.1186/s12906-023-04241-z

**Published:** 2023-11-21

**Authors:** Baydaa Abed Hussein, Isaac Karimi, Namdar Yousofvand

**Affiliations:** Laboratory for Computational Physiology, Department of Biology, Faculty of Science, Razi University, Kermanshah, 67149-67346 Iran

**Keywords:** Myrtle, Acetylcholinesterase, Phyto-nootropics, Insecticides, Terpenes, Sesquiterpenes, *Beta*-bourbonene

## Abstract

**Background:**

*Myrtus communis* L. (MC) has been used in Mesopotamian medicine. Here, the cholinesterase (ChE) inhibitory potential of its methyl alcohol extracts has been investigated and computationally dissected.

**Method:**

The ChE inhibition has been measured based on usual Ellman’s colorimetric method compared to a canonical ChE inhibitor, eserine. Through a deep text mining, the structures of phytocompounds (= ligands) of MC were curated from ChemSpider, PubChem, and ZINC databases and docked into protein targets, AChE (PDB 1EVE) and BChE (PDB 1P0I) after initial in silico preparedness and binding affinity (BA; kcal/mol) reported as an endpoint. The calculation of ADMET (absorption, distribution, metabolism, excretion, and toxicity) features of phytocompounds were retrieved from SwissADME (http://www.swissadme.ch/) and admetSAR software to predict the drug-likeness or lead-likeness fitness. The Toxtree v2.5.1, software platforms (http://toxtree.sourceforge.net/) have been used to predict the class of toxicity of phytocompounds. The STITCH platform (http://stitch.embl.de) has been employed to predict ChE-chemicals interactions.

**Results:**

The possible inhibitory activities of AChE of extracts of leaves and berries were 37.33 and 70.00%, respectively as compared to that of eserine while inhibitory BChE activities of extracts of leaves and berries of MC were 19.00 and 50.67%, respectively as compared to that of eserine. Phytochemicals of MC had BA towards AChE ranging from -7.1 (carvacrol) to -9.9 (ellagic acid) kcal/mol. In this regard, *alpha*-bulnesene, (Z)-gamma-Bisabolene, and *beta*-bourbonene were top-listed low toxic binders of AChE, and (Z)-gamma-bisabolene was a more specific AChE binder. *Alpha*-cadinol, estragole, humulene epoxide II, (a)esculin, ellagic acid, patuletin, juniper camphor, linalyl anthranilate, and spathulenol were high class (Class III) toxic substances which among others, patuletin and *alpha*-cadinol were more specific AChE binders. Among intermediate class (Class II) toxic substances, *beta*-chamigrene was a more specific AChE binder while semimyrtucommulone and myrtucommulone A were more specific BChE binders.

**Conclusion:**

In sum, the AChE binders derived from MC were categorized mostly as antiinsectants (e.g., patuletin and *alpha*-cardinal) due to their predicted toxic classes. It seems that structural amendment and stereoselective synthesis like adding sulphonate or sulphamate groups to these phytocompounds may make them more suitable candidates for considering in preclinical investigations of Alzheimer’s disease.

**Supplementary Information:**

The online version contains supplementary material available at 10.1186/s12906-023-04241-z.

## Background

**Fig. 1:**
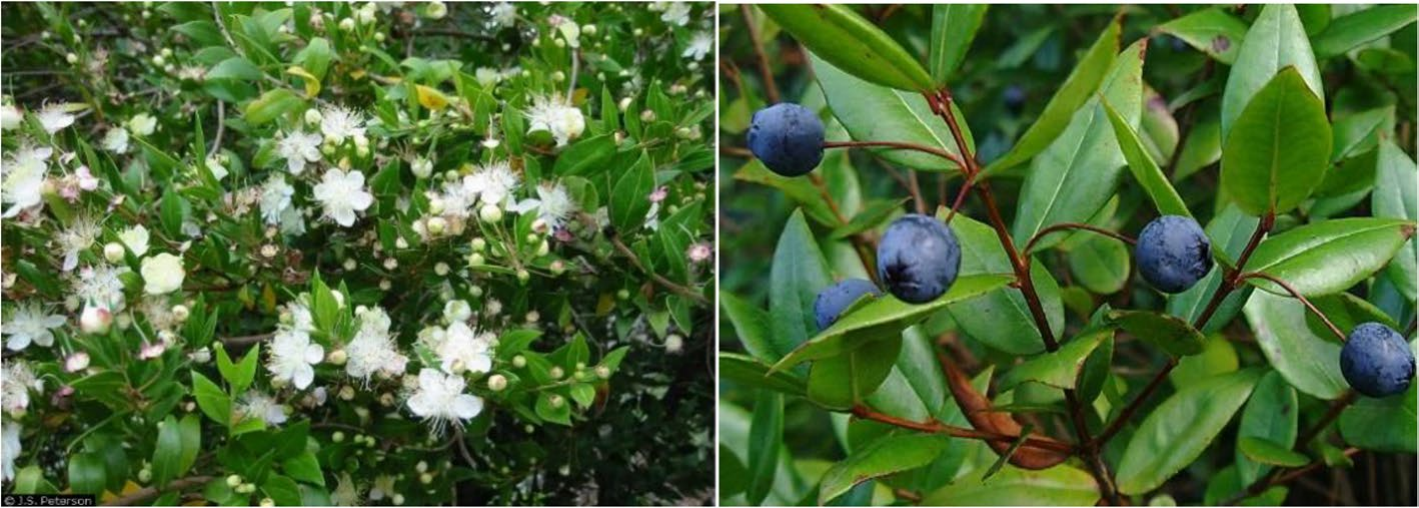
Flowers and fruits of *Myrtus* *communis* L.

*Myrtus communis* L. (MC), Myrtaceae, is known as the common myrtle. It is an aromatic evergreen perennial shrub or small tree 2–3 m high and its branches forming a close full head, thickly covered with leaves. Leaves are small and green and fruits, and berries are small and dark. Flowers are star-like, white or pinkish, and very fragrant (Fig. 1). The round blue-black berry fruit contains several seeds [1]. In this line, Southern Europe, North Africa, and West Asia represent the original countries for growing MC and it has been transferred to South America, Northwestern Himalayas, and Australia and it has been widespread in the Mediterranean region. It is also cultivated in gardens of the North-west Indian region for its fragrant flowers [2]. In ancient Mesopotamian Medicine, it was used for the treatment of fever, headache; skin, bone, heart, digestive, ear, and lung diseases; and gynecological problems [3, 4]. However, there are no comparable studies that pertained to the evidence-based rationale of these aforementioned applications in Mesopotamian Medicine.

The phytochemical screening of myrtle revealed the presence of various classes of chemicals including alkaloids, tannins, phenols, flavonoids, terpenoids, saponins, anthraquinones, proteins, and quinones [5]. Myrtle has many pharmacological and therapeutic effects, such as anti-oxidative, anti-cancer, anti-diabetic, anti-inflammatory, anti-viral, anti-bacterial, anti-fungal, hepatoprotective, and neuroprotective activities [1].

Several previous studies have reported that the essential oil of myrtle has an insecticidal effect [1]. In an experimental study, Tumen et al. [6] have screened the inhibitory effects of dichloromethane, acetone, ethyl acetate, and methanolic extracts of the leaves and fruits of myrtle for three enzymes including acetylcholinesterase (AChE; EC 3.1.1.7), and butyrylcholinesterase (BChE; EC 3.1.1.8) and tyrosinase (EC 1.14.18.1) which are involved in the pathogenesis of neurodegenerative diseases. These extracts were reported to exert moderate AChE and tyrosinase inhibitory effects. BChE inhibition was almost negligible in the case of leaf extracts, whereas the fruit extracts displayed overt inhibition.

The deciphering and designing of cholinesterase (ChE) inhibitors have been pursued by various researchers who want to treat Alzheimer’s disease (AD) or control insects. Therefore, the purpose of this study was to screen the anti-ChE activity of methyl alcohol extract of myrtle and to decipher its possible mechanisms using in silico tools to introduce the best ChE binders.

## Methods

### Materials

All chemicals have been bought from Sigma-Aldrich (St. Louis, MO, USA), otherwise stated. The leaves and berries of myrtle have been collected from Baghdad, Iraq and authenticated by a botanist in our department, and deposited in our herbarium. The leaves and berries were dried in shade, ground, and frozen at -20 ºC for three days and then freeze-dried using a lyophilizer. The resulting powders were extracted using methyl alcohol for one week with sporadic shaking. All extracts were filtered and then concentrated under reduced pressure using a rotary evaporator at 37 ºC and the resulting semisolids were weighed and stored in a refrigerator in airtight cryo-tubes until further use.

### In vitro ChE inhibitory effect

The ChE inhibition has been assayed according to Ellman’s colorimetric method [7]. In brief, the enzymatic reaction mixture containing enzyme (100 μL), Na2HPO4 buffer solution (1000 μL; 50 mM; pH 7.7), and extract (100 μL) was mixed and read at 412 nm then incubated for 10 min at 37 °C. Afterward, 100 μL of AChE substrate (acetylthiocholine iodide) or BChE substrate (butyrylthiocholine chloride), and 100 μL of Ellman's reagent (DTNB; 5,5´-Dithiobis [2-Nitrobenzoic Acid]) have been added and allowed 30 min at 37°C and absorbance was read at 412 nm. These enzymes hydrolyze the substrates to thiocholine and acetic acid and the former reacts to DTNB to produce a yellowish compound, 5-thio-2-nitrobenzoate. All assays were performed three times and eserine or physostigmine, as a reversible ChE inhibitor, was employed as a positive control. The percent inhibition was calculated using the equation: Inhibition (%) = (absorbance of negative control—absorbance of extract)/ (absorbance of negative control) × 100; where, control = total enzyme activity without inhibitor; test = activity in the presence of extract or eserine.

### Statistical analysis

All ChE inhibition assays were performed in three replicates and results were expressed as percentages in comparison to those of positive control, eserine. The comparisons of enzyme inhibitions between beans and leaves were analyzed using Student’s t-test at a statistically significant level of *P* ≤ 0.05 using statistical software IBM (SPSS version 19.0) for Windows.

### Computational methods (in silico)

#### Molecular docking simulation

**Fig. 2:**
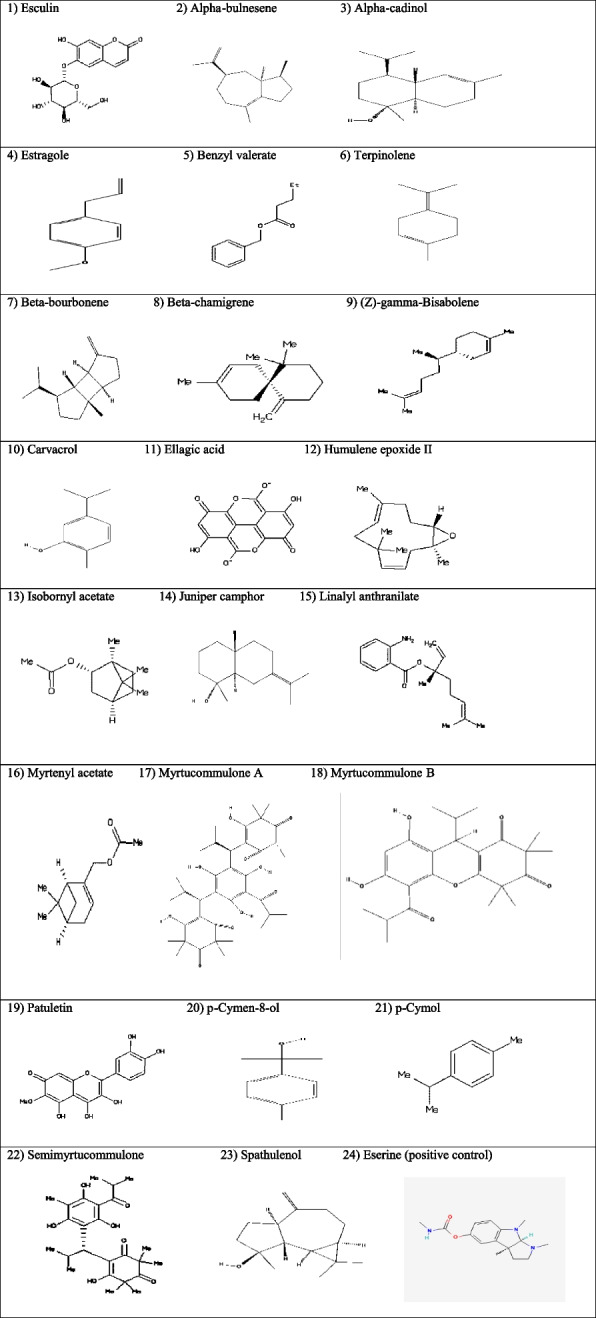
The chemical structure of phytocompounds of *Myrtus communis* L

The structures of phytocompounds (= ligands) of myrtle were harvested from ChemSpider databases (http://www.chemspider.com), PubChem databases (https://pubchem.ncbi.nlm.nih.gov), and ZINC database (http://zinc.docking.org/; Fig. 2). The X-ray crystal structures of protein targets, AChE (1EVE: Pacific electric ray (*Torpedo californica*); [Tax Id: 7787]) and BChE (1P0I: Human (*Homo sapiens*); [Tax Id:9606]) were picked up from Protein Data Bank (PDB; http://www.RCSB.org; [8]), edited, optimized, and trimmed from structural cracks and corruptions in Molegro Virtual Docker machine [9] and Chimera 1.8.1 (http://www.rbvi.ucsf.edu/chimera) before importing into PyRx software version 0.8 [10]. The number of each amino acid that has been reported in edited 1EVE is Torpedo’s number minus one and in edited 1P0I is equivalent to *Homo sapiens’* number minus three for any comparison. The computational results of in silico molecular docking using Vina Wizard were represented as binding affinity (BA; kcal/mol) values.

The docking validation was pursued just for PDB: 1EVE (complexed with donepezil) because no inhibitor is reported in complex structure of 1P0I. The donepezil was trimmed away from its PDB format in Molegro Virtual Docker machine and re-docked in PyRx software. To confirm the accuracy of the docking protocol, the re-docked conformation of donepezil was aligned to the original conformation and the root mean square deviation (RMSD) was calculated. The lower and upper RMSD values obtained for 1EVE were 1.308 and 2.539, respectively while BA of donepezil were -10.4 kcal/mol.

### Cheminformatics

The calculation of ADMET (absorption, distribution, metabolism, excretion, and toxicity) features is at the heart of drug or toxin discovery. The structures of the major phytocompounds of myrtle were retrieved from databases (vide supra). Afterward, their canonical SMILES formats were submitted to AdmetSAR Database (https://lmmd.ecust.edu.Cn/admetsar1) and SwissADME (http://www.swissadme.ch/) and predicted pharmacokinetic parameters including blood–brain barrier (BBB) penetration, human gastrointestinal tract (GIT) absorption, distribution, subcellular localization, metabolism via CYP450 2C9, and human *ether-a-go-go-related gene* (*HERG*) inhibition to screen possible cardiotoxicity have been selected criteria besides Lipinski’s rule of five (RO5) for predicting the drug-likeness or lead-likeness fitness [11]. Moreover, the whole cheminformatics including pharmacokinetics, drug-likeness, and medicinal chemistry friendliness of strong binders of ChE has been presented in Supplementary file S1.

### Bioinformatics

The bioinformatics analysis was conducted through the STITCH platform (http://stitch.embl.de). Explicitly, a component‐target analysis was built by considering the reported phytochemicals of MC and human target proteins. In addition, the STITCH platform has been employed to present ChE-chemicals interactions.

## Results

### In vitro* anti-cholinesterase assay*

The inhibitory activities of AChE of extracts of leaves and berries were 37.33 and 70.00% compared to that of eserine, respectively. The inhibitory BChE activities of extracts of leaves and berries of MC were 19.00 and 50.67% compared to that of eserine, respectively. The parts of the plant showed that inhibition of AChE is greater than that of BChE. In MC, the berries extract has more activity than the leaf extract (*P* ≤ 0.05). Methyl alcohol extract of berries of MC showed noteworthy anti-ChE activity for AChE (70.00%) and BChE (50.67%; *p* = 0.03), while the leaves of MC itself showed less inhibitory activity for AChE (37.33%) and BChE (19.00%; *p* = 0.016), respectively.

### Cheminformatics

We reviewed the previous literature reporting the chemical compositions of MC in Google Scholar and retrieved their chemical information from the most common chemical databases, including ZINC and/or PubChem databases (Fig. 2). The BAs of phytochemicals of MC and protein targets of AChE and BChE have been presented in Tables 1 and 2, respectively.

**Table 1:** In silico molecular docking of major phytochemicals of *Myrtus communis* L. against AChE (PDB code 1EVE)

Ligand-code	Binding affinity (Kcal/mol)	RMSD/UB	RMSD/LB
(A)esculin-3860441 Z	-9.2	2.459	1.819
*alpha*-Bulnesene-85598968 Z	-8.8	4.933	1.298
*alpha*-Cadinol-171679 Z	-8.4	4.293	2.162
Estragole- 8815 P	-6.9	1.527	0.444
Benzyl valerate- 2,515,963 Z	-7.3	1.970	1.203
*beta*-Bourbonene-59778978 Z	-8.7	6.355	3.188
*beta*-Chamigrene-968468 Z	-8.9	4.542	1.309
(Z)-gamma-Bisabolene-2582084	-8.6	1.741	0.807
Carvacrol-967563 Z	-7.1	4.507	1.182
Ellagic acid-86860227 Z	-9.9	4.797	1.043
Humulene epoxide II-9213617 Z	-8.6	3.493	2.046
Isobornyl acetate-388662 Z	-7.4	3.695	2.274
Juniper camphor-5318734 Z	-8.7	7.625	5.874
Linalyl anthranilate-1697404 Z	-8.1	2.992	1.676
Myrtenyl acetate-1850908 Z	-7.3	3.736	2.697
Myrtucommulone A- 44,587,062 P	-7.6	6.654	1.926
Myrtucommulone B- 9,888,014 P	-7.7	3.061	1.989
Patuletin-06483403 Z	-9.0	7.818	1.843
*p*-Cymen-8-ol- 14,529 P	-7.4	1.438	0.040
*p*-Cymol- 968,246 Z	-7.3	4.366	0.916
Semimyrtucommulone-14695529 Z	-7.0	3.936	2.063
Spathulenol- 87,493,019 Z	-8.2	3.662	1.418
Terpinolene- 11,463 P	-7.3	4.324	1.158
Eserine	-6.4	3.333	5.687

*RMSD* Root mean-square deviation is the measure of the average distance between the atoms, *UB* Upper bound, *LB* Lower bound, *Z* Stated for ZINC database version 12 or 15, P stated for PubChem database

**Table 2:** In silico molecular docking of major phytochemicals of *Myrtus communis* L. against BChE (PDB code 1P0I)

Protein–Ligand-code	Binding Affinity (Kcal/mol)	RMSD/UB	RMSD/LB
(A)esculin-3860441 Z	-8.8	7.007	3.81
*alpha*-Bulnesene-85598968 Z	-7.4	2.641	1.306
*alpha*-Cadinol-171679 Z	-7.3	5.44	2.671
Estragole- 8815 P	-5.9	5.173	1.88
Benzyl valerate- 2,515,963 Z	-6.3	6.914	5.213
*beta*-Bourbonene-59778978 Z	-7.4	4.475	1.306
*beta*-Chamigrene-968468 Z	-7.5	4.825	1.754
(Z)-gamma-Bisabolene-2582084	-6.9	3.494	1.744
Carvacrol-967563 Z	-6.1	4.465	1.805
Ellagic acid-86860227 Z	-9.4	4.012	1.331
Humulene epoxide II-9213617 Z	-7.5	21.435	20.226
Isobornyl acetate-388662 Z	-6.3	4.083	1.91
Juniper camphor-5318734 Z	-7.8	4.495	1.579
Linalyl anthranilate-1697404 Z	-7.6	1.723	1.177
Myrtenyl acetate-1850908 Z	-6.3	5.278	2.707
Myrtucommulone A- 44,587,062 P	-8.8	9.388	4.365
Myrtucommulone B- 9,888,014 P	-7.4	22.965	20.514
Patuletin-06483403 Z	-7.9	7.575	1.788
*p*-Cymen-8-ol- 14,529 P	-6.6	1.438	0.013
*p*-Cymol- 968,246 Z	-6.1	4.528	0.919
Semimyrtucommulone-14695529 Z	-9.3	3.496	1.203
Spathulenol- 87,493,019 Z	-7.5	3.691	1.721
Terpinolene- 11,463 P	-6.1	4.439	0.934
Eserine	-7.5	3.602	4.474

*RMSD* Root mean-square deviation is the measure of the average distance between the atoms, *UB* Upper bound, *LB* Lower bound

**Fig. 3:**
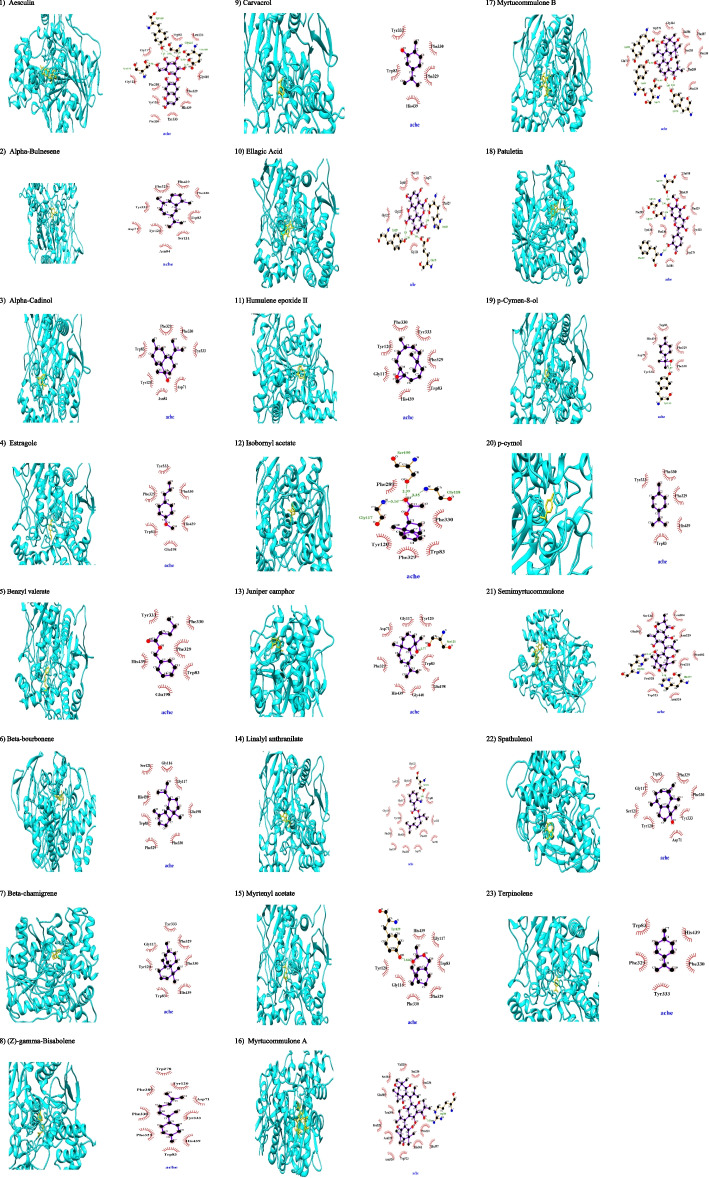
Molecular docking of phytochemicals of *Myrtus communis* L. against AChE (PDB code 1EVE; in cyan color). Hydrogen bonds are indicated by dashed lines between the atoms involved while hydrophobic interactions are symbolized by a semicircle with spokes radiating towards the ligand atoms they interact

**Fig. 4:**
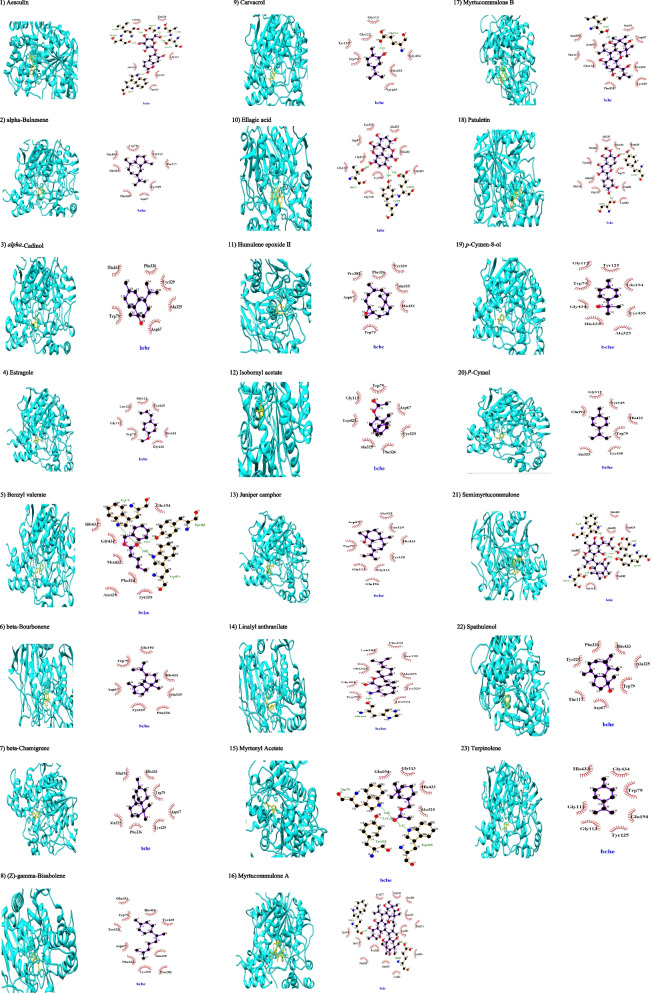
Molecular docking of phytochemicals of *Myrtus communis* L. against BChE (PDB code 1P0I; in cyan color). Hydrogen bonds are indicated by dashed lines between the atoms involved while hydrophobic interactions are symbolized by a semicircle with spokes radiating towards the ligand atoms they interact

In the present study, ellagic acid as a polyphenol has been docked to AChE via hydrogen bonds with Glu198 residue of anionic subsite (AS; vide infra) and His439 residue of acylation site and with the hydroxyl group of Tyr129 and via hydrophobic interactions with amino acid residues of PAS (Asp71) and AS (Trp83 and Phe329) and oxyanion hole (OH; vide infra) (Gly117) (Fig. 3). In addition, it docked with BChE via hydrogen bonds with Glu194 and Tyr125 residues of AS and with the hydroxyl group of Thr117 and through hydrophobic interactions with amino acid residues in peripheral anionic site (PAS; vide infra) (Tyr329 and Asp67) and AS (Ala325 and Trp79) and acylation site (His433) of BChE (Fig. 4). Ellagic acid has accepted ADMET properties and has not any violations against RO5 (Table 3; see Supplementary file 1). However, SwissADME did not support its BBB penetration. The chemoinformatics and bioinformatics of the binders of ChE among phytochemicals of MC have been curated by SwissADME (see Supplementary file 1). However, ellagic acid has a heterocyclic ring with complex substituents with no sulphonate or sulphamate groups in the category of class high (Class III) toxic compound suitable to be considered as an insecticidal botanical agent.

**Table 3:** In silico ADMET properties of phytochemicals of *Myrtus communis* L. by admetSAR

Compound	BBB	HIA	CYP inhibition/substrate	Subcellular localization	HERG Inhibition
(A)Esculin	0.6058	0.5202	Non-substrate/ Non-inhibitor	Mitochondria	Weak
*alpha*-Bulnesene	0.9723	0.9940	Non-substrate/ Non-inhibitor	Lysosome	Weak
*alpha*-Cadinol	0.9455	1.0000	Non-substrate/ Non-inhibitor	Lysosome	Weak
Estragole	0.9383	0.9939	Non-substrate/ inhibitor	Mitochondria	Weak
Benzyl valerate	0.9739	1.0000	Non-substrate/ Non-inhibitor	Plasma membrane	
*beta*-Bourbonene	0.9612	0.9934	Non-substrate/ Non-inhibitor	Lysosome	Weak
*beta*-Chamigrene	0.9554	0.9875	Non-substrate/ Non-inhibitor	Lysosome	Weak
(Z)-*gamma*-Bisabolene	0.9399	0.9934	Non-substrate/ Non-inhibitor	Lysosome	Weak
Carvacrol	0.9381	0.9955	Non-substrate/ Non-inhibitor	Mitochondria	Weak
Ellagic acid	0.5641	0.7199	Non-substrate/ Non-inhibitor	Mitochondria	Weak
Humulene epoxide II	0.9674	1.0000	Non-substrate/ inhibitor	Lysosome	Weak
Isobornyl acetate	0.9719	0.9969	Non-substrate/ Non-inhibitor	Mitochondria	Weak
Juniper camphor	0.9455	1.0000	Non-substrate/ Non-inhibitor	Lysosome	Weak
Linalyl anthranilate	0.9014	0.9669	Non-substrate/ Non-inhibitor	Lysosome	Weak
Myrtenyl acetate	0.9002	0.9950	Non-substrate/ Non-inhibitor	Lysosome	Weak
Myrtucommulone A	0.6582	0.9790	Non-substrate/ inhibitor	Mitochondria	Weak
Myrtucommulone B	0.5297	0.9687	Non-substrate/ inhibitor	Mitochondria	Weak
Patuletin	0.8171	0.9482	Non-substrate/ Non-inhibitor	Mitochondria	Weak
p-Cymen-8-ol	0.9741	0.9944	Non-substrate/ Non-inhibitor	Mitochondria	Weak
p-Cymol	0.9677	0.9960	Non-substrate/ Non-inhibitor	Lysosome	Weak
Semimyrtucommulone	0.6582	0.9790	Non-substrate/ inhibitor	Mitochondria	Weak
Spathulenol	0.9648	0.9913	Non-substrate/ inhibitor	Lysosome	Weak
Terpinolene	0.9299	0.9963	Non-substrate/Non-inhibitor	Lysosome	Weak

*Abbreviations ADMET* Absorption, distribution, metabolism, and excretion-toxicity, *BBB* Blood–brain barrier penetration, *HIA* Human intestinal absorption, *CYP* Cytochrome P, *HERG Human ether-a-go-go-related* genes inhibition

The Phe287 residue of the acyl pocket (AP) interacted with esculin via hydrogen bond while Tyr333, Tyr120, Asp71, Trp69, and Trp278 residues of PAS and Phe289 and Phe330 residues of AP and Phe329 residues of AS interacted hydrophobically with esculin (Fig. 3). It docked with BChE via hydrogen bonding with Trp79 residues of AS and Ser195 residues of acylation site and Leu283 residues of AP and with the hydroxyl group of Ser284, Gly114, and Ala196 and interacted hydrophobically with Ala325 (Fig. 4). Based on ADMET results, it can be absorbed from GIT and crossed BBB properly (Table 3; see Supplementary file 1). Although it has not any violations of RO5 of drug-likeness (see Supplementary file 1) and it was the best AChE binder after ellagic acid (Table 1). This heterocyclic lactone or cyclic diester is categorized as a class high (Class III) toxic substance and would be a suitable antiinsectant rather than anti-AD.

*Alpha*-bulnesene, α-guaiene, is a sesquiterpene that docked with AChE via hydrophobic interactions using amino acid residues of PAS (Tyr333, Tyr120 and Asp71), AS (Trp83 and Phe329), AP (Phe330) and catalytic site (His439) (Fig. 3). It has been docked to BChE via hydrophobic interactions with PAS (Asp67 and Tyr329) and AS (Trp79 and Phe326) and catalytic site (His433) (Fig. 4). Based on ADMET properties, it has the ability to pass BBB and GIT (Table 3; see Supplementary file 1). This common terpene is categorized as a class low (Class I) toxic substance. Recently, possible ChE inhibitory potential and nematicidal properties of *alpha*-bulnesene have been proposed [12] and since this compound has MLOGP > 4.15 which violates Lipinski’s RO5, it would be an appropriate antiinsectant rather than anti-AD (see Supplementary file 1).

*Alpha*-cadinol has been docked to AChE through hydrophobic interactions with PAS (Tyr333, Tyr120, and Asp71) and AS (Trp83 and Phe329), and AP (Phe330) (Fig. 3). It also docked with BChE through hydrophobic interactions with amino acid residues in AS (Trp79, Phe326, and Ala325) and PAS (Asp67 and Tyr329) and catalytic site (His433) (Fig. 4). According to the results of the ADMET assay, it has BBB permeation and GIT absorption (Table 3; see Supplementary file 1). This sesquiterpenoid, cadinane, has not any sulphonate or sulphamate groups and is considered a class high (Class III) toxic substance, while it has not any violations against RO5 (see Supplementary file). It seems that alpha-cadinol cannot be considered as an anti-AD agent and its insecticidal potential would be more reliable.

Estragole showed the weakest BAs for ChE through employing hydrophobic interactions with the catalytic site and AS among all reported compounds of MC while its ChE inhibitory property has been documented ([13]; Figs. 3 and 4). According to the results of the ADMET assay, it penetrates BBB and GIT very efficiently and this benzene derivative is categorized as a class high (Class III) toxic substance with possible antiinsectant activity (Table 3; see Supplementary file 1).

*Beta*-bourbonene as a sesquiterpene has been docked into AChE via hydrophobic interactions with amino acid residues of AS (Trp83, Phe329, and Glu198), AP (Phe330), OH (Gly117), and catalytic site (His439) (Fig. 3). It has been docked to BChE via hydrophobic interactions with PAS (Asp67 and Tyr329) and AS (Phe326, Trp79, and Ala325) and catalytic site (His433). Based on ADMET properties, it can pass BBB and GIT (Table 3; see Supplementary file 1. This common terpene is categorized as a class low (Class I) toxic substance while blocking an array of cytochromes involved in xenobiotics metabolism. It has one violation against Lipinski (Pfizer) filter of RO5 and it would be considered an antiinsectant.

*Beta*-chamigrene is a carbobicyclic sesquiterpene that has been docked to AChE via hydrophobic interactions using amino acid residues of PAS (Tyr333, Tyr120), OH (Gly117), AS (Trp83 and Phe329), AP (Phe330) and catalytic site (His439) (Fig. 3). In addition, it docked with BChE via hydrophobic interactions with AS (Ala325, Trp79, and Phe326) and PAS (Asp67 and Tyr329) and catalytic site (His433). Based on ADMET properties, it has the ability to pass BBB and GIT based on AdmetSAR, while SwissADME did not support its brain permeation. It has one violation against RO5 and insecticidal properties would be more possible than its anti-AD potential (Table 3; see Supplementary file 1). It is categorized as a class intermediate (Class II) toxic substance because it is a monocycloalkanone or a bicyclocompound.

(Z)*-gamma-*Bisabolene is sesquiterpene that docked to AChE via hydrophobic interactions with PAS (Tyr333, Trp278, Asp71, and Tyr120), AS (Trp83 and Phe329), AP (Phe330), and catalytic site (His439) (Fig. 3). It has been docked to BChE via hydrophobic interactions with AS (Ala325, Trp79, and Phe326), PAS (Asp67 and Tyr329), and catalytic site (His433) (Fig. 4). According to the results of ADMET properties, it can be crossed BBB and GIT, and localization in the lysosome (Table 3; see Supplementary file 1). The total polar surface area of (Z)*-gamma-*bisabolene is zero and this violation against RO5 makes this molecule unsuitable for oral bioavailability and structural functionalization is required to hasten its bioavailability. It is monocarbocyclic with simple substituents and therefore categorized as a class low (Class I) toxic substance suitable for designing bio-inspired antiinsectants.

Carvacrol, cymophenol, is a monoterpenoid phenol, which has been docked into catalytic acylation site (His439), AS (Phe 329), and acyl pocket (Phe331) of AChE stronger than AS (Trp79, Tyr125) of BChE (Figs. 3 and 4). This aromatic hydrocarbon has been categorized as a class low (Class I) toxic substance with accepted and reliable ADMET endpoints like high GIT absorption and good BBB permeation (Table 3; see Supplementary file 1). In conclusion, carvacrol can be a lead molecule to be considered an anti-AD or antiinsectant.

Humulene epoxide II belongs to epoxides and it has been docked into AChE via hydrophobic interactions with amino acid residues in AP (Phe330) and PAS (Tyr333 and Tyr120) and AS (Trp83 and Phe329) and OH (Gly117) and acylation site (His439) (Fig. 3). In this regard, humulene epoxide II has been docked into BChE through hydrophobic interactions using amino acid residues in PAS (Tyr329) and AS (Ala325, Phe326, and Trp79) (Fig. 4). Our ADMET prediction showed that it could be crossed from GIT and BBB properly without any violations against RO5 while this heterocyclic hydrocarbon has been categorized as a class high (Class III) toxic substance (Table 3; see Supplementary file 1) and it should be considered as an insecticidal lead molecule. 

Isobornyl acetate, a bicyclic monoterpenoid, has been docked with the AChE catalytic acylation site (Ser199) and acylation pocket (Phe288) and Gly118, Glyc117 of the anionic site through hydrogen bonds and it interacted with Phe329 and Trp83 of AS through hydrophobic interactions (Fig. 3). Isobornyl acetate has been also interacted with BChE using hydrophobic interactions with an anionic site weaker than that of AChE (Fig. 4). In a seminal review, Wojtunik-Kulesza and colleagues [14] introduced a bunch of monoterpenes including isobornyl acetate, camphor, carvacrol, bornyl acetate, and myrtenal, among others as phytocompounds useful to treat AD Symptoms in experimental model studies. Based on the computational findings of ToxTree, this phytocompound is readily hydrolyzed to a common terpene, possesses open-chain aliphatic with some functional groups, and is finally categorized as a class low (Class I) toxic flavoring agent with accepted ADMET which obeys RO5 (Table 3; see Supplementary file 1). Therefore, it should be considered an anti-AD lead molecule.

Juniper camphor is a hardly soluble sesquiterpenoid compound which has been docked into AChE through hydrogen bonds with the hydroxyl group of Ser121 and through hydrophobic interactions with PAS (Asp71 and Tyr120) and OH of the catalytic triad (Gly117), AS (Phe329 and Trp83) and catalytic site (His439) (Fig. 3). It has been docked with BChE via hydrophobic interactions with AS (Ala325 and Tyr125), PAS (Asp67 and Tyr329), and catalytic site (His433) (Fig. 4). Juniper camphor has the ability to penetrate BBB and cross GIT while it is a weak HERG inhibitor. This aliphatic homopolycyclic compound has not any sulphonate or sulphamate groups and is considered a class high (Class III) toxic substance, although it follows RO5. Therefore, it should be considered an insecticidal lead molecule.

Benzyl valerate showed stronger BA with AChE rather than BChE. However, it bounded into BChE via hydrogen bonds (Figs. 3 and 4). It has been lodged into AS of AChE via hydrophobic interactions. This molecule can be absorbed from GIT and transferred from BBB efficiently and follow RO5 and is considered an anti-AD flavoring agent. This aromatic hydrocarbon has been categorized as a class low (Class I) toxic compound (see Supplementary file 1).

Linalyl anthranilate as an aromatic monoterpenoid compound has been docked AChE (BA of -8.1 kcal/mol) using hydrogen bonds with the hydroxyl group of Ser121 and through hydrophobic interactions with AS (Trp83 and Phe329), PAS (Tyr333, Asp71, and Tyr120), OH (Gly117), AP (Phe330), and acylation site (His439 and Ser199) (Fig. 3). It has a BA of -7.6 kcal/mol with BChE using hydrogen bonding with the catalytic site (His433) and through hydrophobic interactions with amino acid residue of AS (Trp79 and Ala325) and AP and AP (Leu283) (Fig. 4). It can be absorbed from GIT, crossed BBB, and localized in lysosome while it is a week inhibitor of HERG while it followed RO5. Based on computational results of Toxtree, linalyl anthranilate is categorized as high (Class III) toxic substance possesses rings with reactive functional substituents without Na, K, Ca sulphonate and sulphamate salts which decrease its toxicity (Table 3; see Supplementary file 1). Consequently, it should be considered an insecticidal lead molecule rather than an anti-AD agent.

Myrtenyl acetate as a bicyclic monoterpenoid has interacted with the catalytic site of AChE (His440) and AP (Phe330) through hydrophobic interactions while it has been docked through using hydrogen bonds with OH and AS of BChE (Figs. 3 and 4). This phytocompound has tropism to the lysosome for metabolism and penetrates BBB and crosses GIT while it is a weak HERG inhibitor that follows RO5 (Table 3; see Supplementary file 1). It can readily be hydrolyzed to a common terpene and it is an aliphatic with some functional groups and can be classified as a class low (Class I) toxic substance suitable to be considered as an anti-AD lead molecule.

Myrtucommulone A as an aromatic ketone has been docked with AChE through hydrogen bonds with the hydroxyl group of Arg288. It has been docked to BChE via hydrogen bonds with the hydroxyl group of Glu399 and Trp516 (Figs. 3 and 4). This compound bears an aromatic ring with complex substituents and has been allocated as a class intermediate (Class II) toxic substance with an accepted ADMET (Table 3; see Supplementary file 1). Consequently, it should be considered as an insecticidal lead molecule rather than an anti-AD agent since it has one violation against RO5.

Myrtucommulone B docked with AChE through hydrogen bonds with amino acid residues of PAS (Tyr333, Tyr120, Tyr69, and Asp71) and via hydrophobic interactions with amino acid residues of PAS (Trp278) and A (Phe329) (Fig. 3). It has been docked to BChE via hydrogen bonds with the hydroxyl group of Ile66 and via hydrophobic interactions with PAS (Asp67 and Tyr329) and AS (Phe326) (Fig. 4). The ADMET assay showed it is a moderately soluble compound that cannot penetrate BBB appropriately while crossing GIT efficiently in accordance with RO5 (Table 3; see Supplementary file 1). Myrtucommulone has not a sufficient number of sulphonate or sulphamate groups and is categorized as a class high (Class III) toxic substance and it may have insecticidal properties.

Patuletin has been docked to catalytic triad of AChE using hydrogen bonds (Ser199, Gly117, and Gly118) and with AP (Phe287) while Tyr333, Tyr120, and Trp278 residues of PAS and Phe330 residue of AP and Phe329 and Glu198 residues of AS interacted hydrophobically with patuletin (Fig. 3). In addition, it has been docked into BChE via hydrogen bonds using Tyr329 residues of PAS and using hydroxyl group of Ser284 and hydrophobic interactions which interacted with amino acid residues in AP (Leu283) and AS (Ala325 and Trp79) (Fig. 4). The ADMET assays showed that it can be absorbed orally without BBB permeation while it is a weak inhibitor of HERG (Table 3; see Supplementary file 1). This aliphatic homopolycyclic compound has not any sulphonate or sulphamate groups and is considered a class high (Class III) toxic substance and can be considered as an antiinsectant.

*p*-Cymen-8-ol has been docked to catalytic triad of AChE using hydrophobic interaction (His439) and with AS (Trp83, Phe329) while Tyr120 has been constructing hydrogen bond with its aliphatic part (Fig. 3). In addition, it docked with BChE via hydrophobic interactions using His438, Tyr125, and Ala328 residues of AS (Fig. 4). The ADMET assays showed that it can be absorbed orally and crossed BBB while it is a weak inhibitor of HERG (Table 3; see Supplementary file 1). This aromatic hydrocarbon has a complex substituted ring without any sulphonate or sulphamate groups and is considered a class high (Class III) toxic substance and can be considered an antiinsectant.

*p*-Cymol, an alkylbenzene related to a monoterpene, has been docked using hydrophobic interaction to catalytic triad (His439), AS (Phe329), PAS (Tyr333), and AP (Phe330) of AChE (Fig. 3). In addition, it docked with BChE via hydrophobic interactions with Trp82, Tyr125, and Ala328 residues of AS (Fig. 4). The ADMET assays showed that it can be absorbed orally and crossed BBB while it is a weak inhibitor of HERG (Table 3; see Supplementary file 1). This aromatic hydrocarbon has a substituted ring and is considered a class low (Class I) toxic substance. In spite of *p*-cymen-8-ol which followed RO5, *p*-cymol would be a less toxic anti-AD agent.

Semimyrtucommulone as an oligomeric, nonprenylated acylphloroglucinol has been docked to AChE via hydrogen bonds with the hydroxyl group of His397 and His361 (Fig. 3). It also docked with BChE through hydrogen bonds with PAS (Asp67 and Tyr329) and AS (Trp79) and with the hydroxyl group of Thr117 and via hydrophobic interactions with Ala325 (Fig. 4). Semimyrtucommulone can be absorbed from GIT, crossed BBB scarcely, and localized in mitochondria while it is a weak inhibitor of HERG (Table 3; see Supplementary file 1). This aromatic compound bearing rings with complex substituents is listed as a class intermediate (Class II) toxic substance while following RO5 and can be considered as an antiinsectant.

Spathulenol as tricyclic sesquiterpene alcohol has been hydrophobically interacted with PAS (Tyr333, Asp71 and Tyr120), AP (Phe330), AS (Trp83 and Phe329) and acylation site (His439) of AChE (Fig. 3). In this line, it has been docked to BChE through hydrophobic interactions with amino acid residues in PAS (Tyr329 and Asp67) and AS (Ala325, Phe326 and Trp79) and acylation site (His433) (Fig. 4). Spathulenol can be absorbed vastly from GIT, crossed overtly BBB, while it is a weak inhibitor of HERG (Table 3; see Supplementary file 1). This aliphatic homopolycyclic compound has not any sulphonate or sulphamate groups and is considered a class high (Class III) toxic substance while following RO5 and can be considered as an antiinsectant.

Terpinolene, as a cyclic monoterpene hydrophobically interacted with PAS (Tyr333), AS (Trp83, Phe330), and the acylation site (His439) of AChE while it hydrophobically interacted with the acylation site (Phe433), AS (Trp79, Tyr125) and OH (Gly113) of BChE (Figs. 3 and 4). This monocarbocyclic phytocompound has simple substituents and is listed as a class low (Class I) toxic substance with reliable ADMET that followed RO5 and can be considered as an anti-AD agent (Table 3; see Supplementary file 1).

### Bioinformatics

**Fig. 5:**
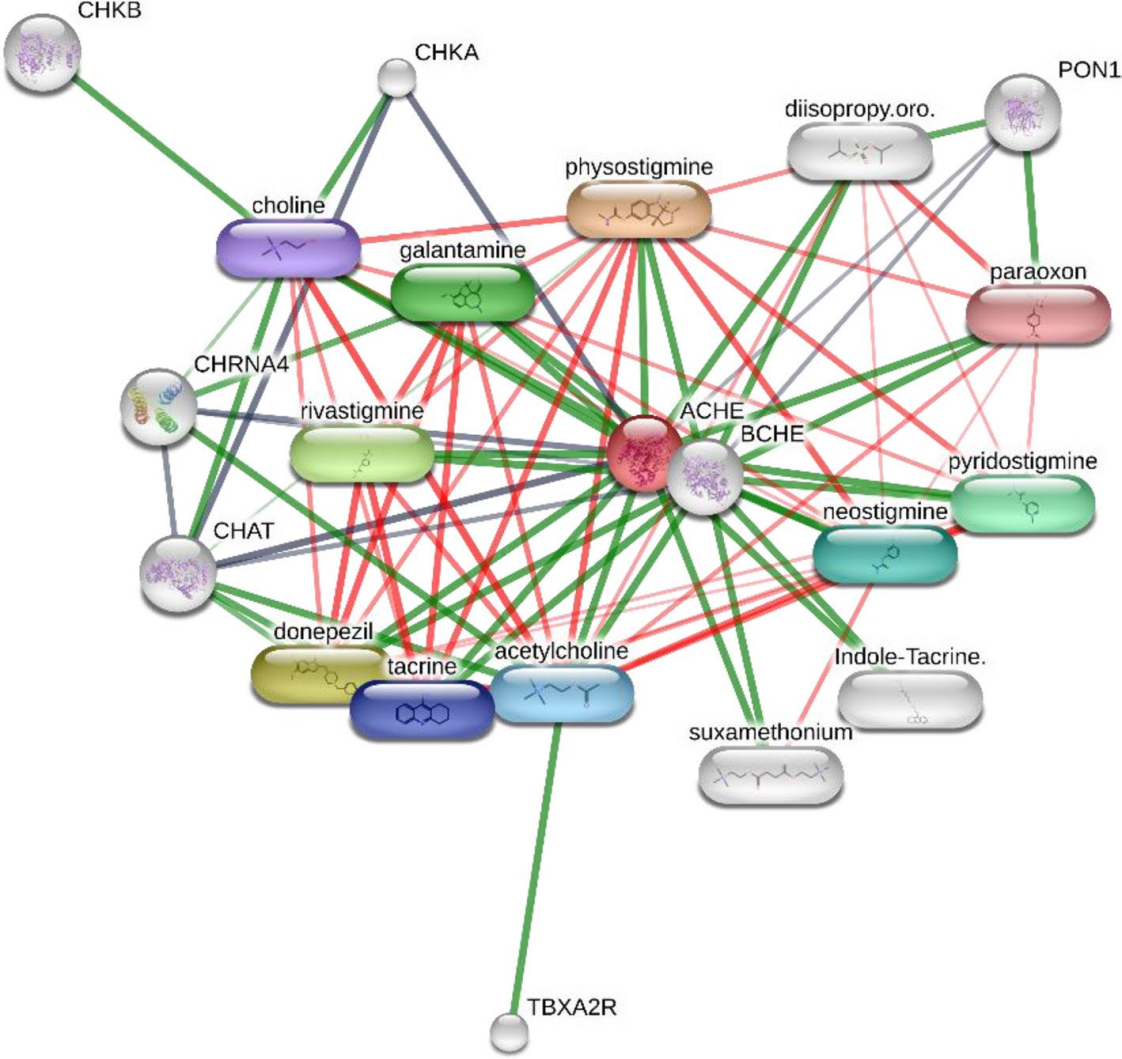
Cholinesterase (PDB ID 3LII)-components analysis conducted on the STITCH bioinformatics platform to dissect possible interactions between myrtle phytochemicals and major players of cholinergic system in *Homo sapiens* (see text or STITCH website for more explanation)

**Fig. 6:**
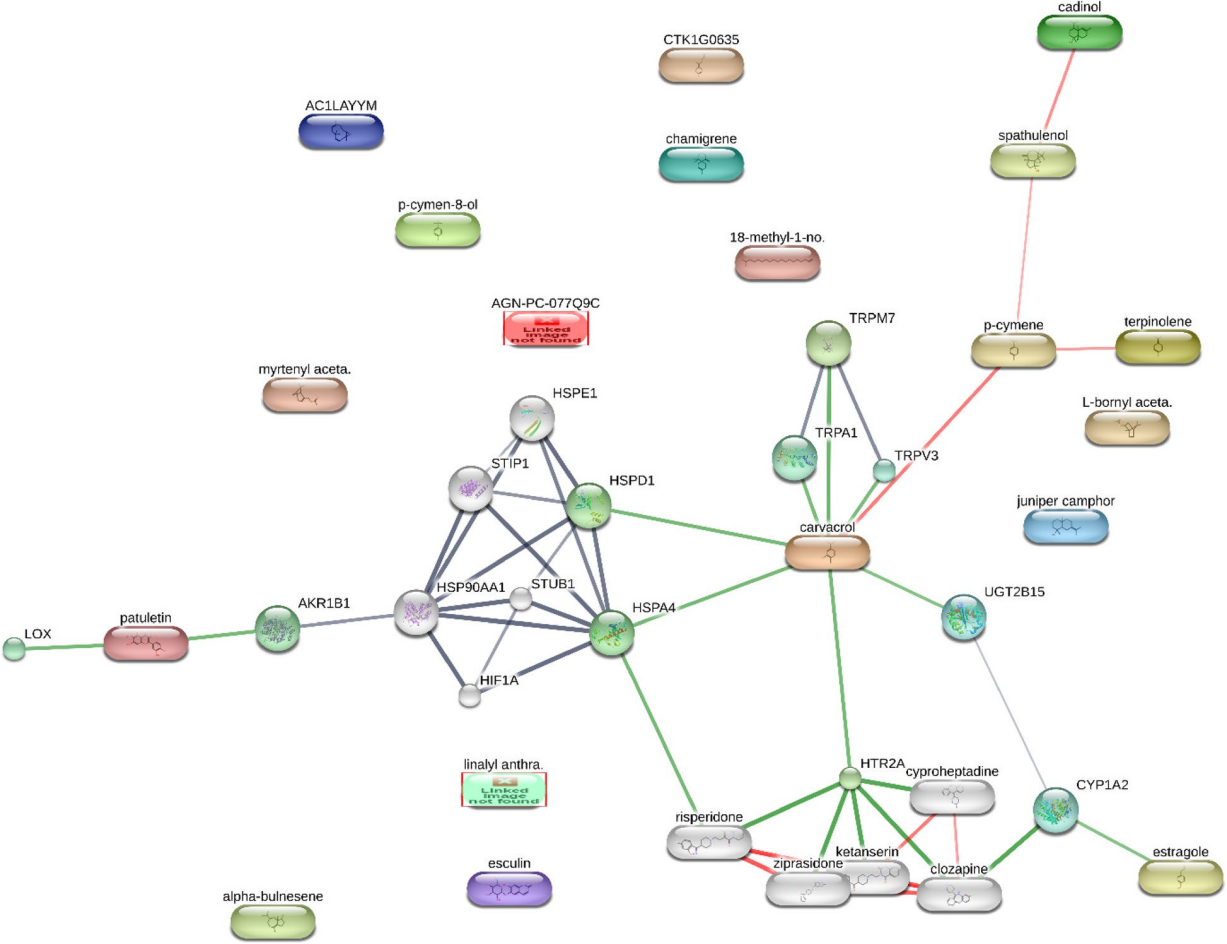
Confidence view of components–targets analysis constructed through the bioinformatics STITCH platform that predicted interactions between phytochemicals of myrtle and human multiple protein classes, including aldo–keto reductase family 1 (AKR1B1), heat shock proteins (HSP), hypoxia inducible factor (HIF), stress-induced-phosphoprotein 1 (SIP1), STIP1 homology and U-box containing protein 1 (STUB1), 5-hydroxytryptamine (serotonin) receptor 2A (HTR2A), lysyl oxidase (LOX), transient receptor potential cation channel, subfamily A (TRPA1), UDP glucuronosyltransferase 2 family, polypeptide B15 (UGT2B15), kinases and cytochromes

**Fig. 7:**
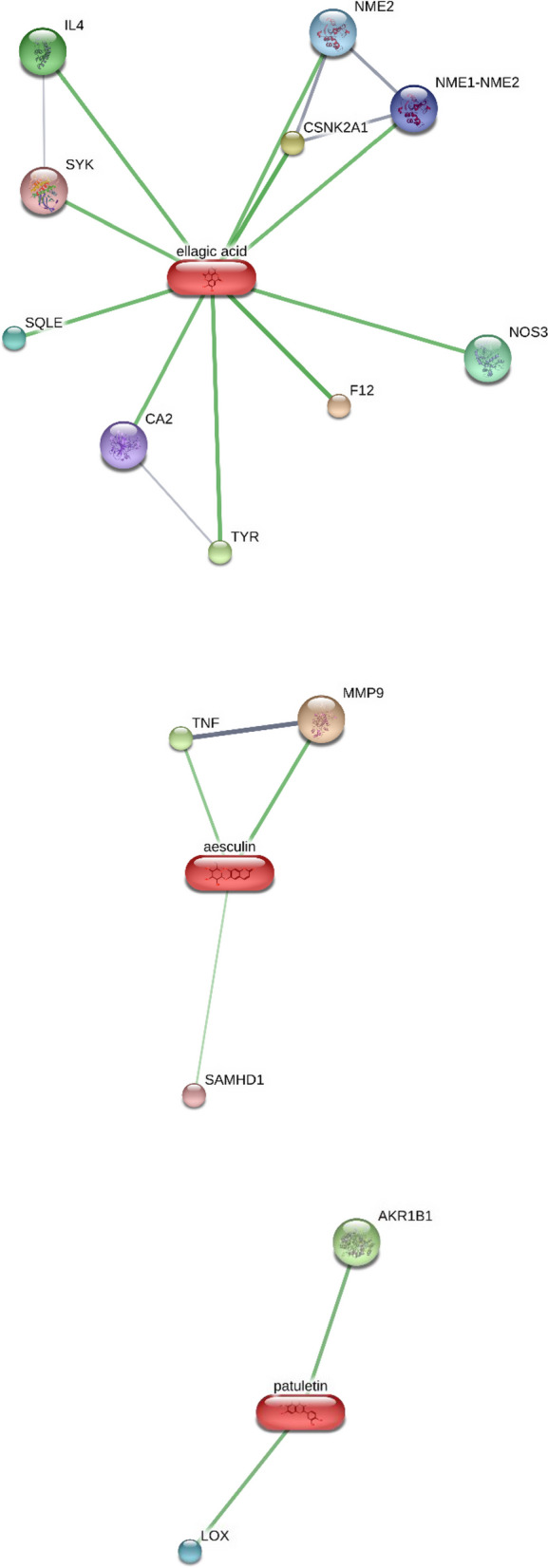
Targets–components analysis conducted on the STITCH bioinformatics platform for unraveling putative interactions between the strongest binders of cholinesterase derived from myrtle and putative proteins. Carbonic anhydrase II CA2, casein kinase 2, *alpha* 1 polypeptide (CSNK2A1), carbonic anhydrase II (CA2), casein kinase 2, *alpha* 1 polypeptide (CSNK2A1), NME1-NME2 readthrough, NME/NM23 nucleoside diphosphate kinase 2 (NME2); coagulation factor XII (Hageman factor); Factor XII (F12), nitric oxide synthase 3 (NOS3), squalene epoxidase (SQLE). spleen tyrosine kinase (SYK), tyrosinase (TYR), interleukin 4 (IL4), matrix metallopeptidase 9 (MMP9), tumor necrosis factor (TNF), SAM domain and HD domain 1 (SAMHD1), aldo–keto reductase family 1, member B1 (AKR1B1), lysyl oxidase (LOX)

As shown in Fig. 5, AChE and BChE were linked together at the heart of a heterogeneous network. In this context, these ChEs were linked to three categories of drugs, namely; anti-AD, antiinsectants, and key players of cholinergic system, including proteins and chemicals. Proteins that are involved in various aspects of the cholinergic system in this network with a low enrichment score of (protein–protein interaction p-value:0.293) including choline O-acetyltransferase (CHAT), choline kinase alpha (CHKA), cholinergic receptor, nicotinic, alpha 4 (neuronal; CHRNA4), paraoxonase 1 (PON1), thromboxane A2 receptor (TBXA2R), and choline kinase beta (CHKB). In addition, acetylcholine, choline chloride, and suxamethonium chloride, also known as suxamethonium or succinylcholine, a nicotinic acetylcholine receptor agonist, are natural or synthetic ligands or precursors for cholinergic receptors. Organophosphorus insecticides (OPs as AChEIs) which have been curated from STITCH derived target-chemicals network include diisopropylfluorophosphate (DFP; Diisopropyl fluorophosphate (DFP, DIFP, diisopropyl phosphorofluoridate), paraoxon, physostigmine, pyridostigmine, and rivastigmine. In this line, AChEIs are used as anti-AD agents, including indole-tacrine heterodimer 6, donepezil, galantamine, neostigmine, and tacrine. The component–target analysis of MC shows that our study is the first to demonstrate that MC possesses anti-ChE activity because AChE and BChE are not curated in Figs. 6 and  7.

## Discussion

Inconsistent with our findings, Tumen et al. [6] showed that the leaf extracts of MC were ineffective against BChE, while the berry extracts displayed inhibition between 21.83 ± 3.82% and 36.80 ± 2.00% and they proposed in vitro neuroprotective effects of myrtle which needs more experiments to prove this claim. Our findings also showed that berries of MC had dual inhibitory effects against AChE and BChE stronger than its leaves. However, its leaves had more inhibitory effects against AChE. In another study, the ChE inhibitory effects of methanolic extract of MC and its various fractions, especially chloroform fraction have been reported 81 to 91% against AChE and 79 to 94.5% against BChE [15]. The ChE inhibitory effects of the essential oils obtained from the leaves of MC have been reported and, besides their antioxidative and neuroprotective effects, the presence of α-pinene, 1,8-cineole, linalool, limonene, and myrtenyl acetate as major constituents has been confirmed [16]. Due to ChE inhibitory effects, traditional and modern Myrtle Shampoo like Sehat®, IRAN may be considered antiinsectants because ACh also is an essential neurotransmitter involved in the muscle contractions of insects, and ChE inhibitors may lead to ACh build up in the synaptic clefts of cholinergic neurons or neuromuscular junction which fulminated to muscle paralysis of insects [17]. Surprisingly, MC extract is a powerful antibacterial and antiseptic plant that helps eliminate or reduce the growth of microorganisms on the scalp [18].

In the present study, our findings clearly showed that ellagic acid, esculin, patuletin, *beta*-chamigrene, *alpha*-bulnesene, *beta*-bourbonene, and (Z)-*gamma*-Bisabolene have more negative BA with AChE at -9.9, -9.2, -9.0, -8.9, -8.8, -8.7, and -8.6 kcal/mol, respectively. In addition, several phytocompounds present in MC have negative BA lesser than -6.0 kcal/mol, therefore, they may have more inhibitory activity for AChE. In addition, our findings showed that ellagic acid, semimyrtucommulone, esculin, myrtucommulone A, patuletin, and Juniper camphor have a more negative BA with BChE at -9.4, -9.3, -8.8, -8.8, -7.9, and -7.8 kcal/mol, respectively. While *beta*-chamigrene, humulene epoxide II, and spathulenol showed the same negative BA at -7.5 (kcal/mol) for AChE as compared with *alpha*-bulnesene, *alpha*-cadinol, *beta*-bourbonene, and myrtucommulone B which have same negative BA at -7.4 (kcal/mol) and they have more potential to be considered as binders of BChE.

The base of the hydrophobic active gorge of AChE and BChE acts as a catalytic region juxtaposed to a choline-binding site [19]. Although AChE is specific to ACh hydrolysis, BChE is promiscuous to its substrates and metabolizes diverse molecules [20]. The catalytic acylation site of AChE is furnished with Ser200, His440, and Glu327 residues, whilst the catalytic triad in BChE contains Ser198, His438, and Glu325 residues [21]. The oxyanion hole (OH) consists of Gly118, Gly119, and Ala201 residues [22] in AChE while the OH subsite is located juxtaposed to the choline-binding site and contains Gly116, Gly117 and Ala199 residues in BChE [23]. Another subsite of AChE is anionic subsite (AS) which contains aromatic residues (Trp84 and Phe330) and Glu199 in AChE [24] whilst AS in BChE contains Trp82, Tyr128, Phe329, and Ala328 residues [25]. The dissimilarity between AChE and BChE lies in the acyl pocket (AP) and the peripheral anionic site (PAS; [26]). The AP of AChE includes Trp233, Phe288, Phe290, and Phe331 residues [27] while the AP of BChE contains Leu286 and Val288 residues. The PAS of AChE includes Tyr70, Asp72, Tyr121, Trp279, and Tyr334 residues [28] while the PAS of BChE contains Tyr332 and Asp70 residues [23].

Further, in vitro dose–response experiments and posology are requested to decipher the putative anti-ChE activity of phytocompounds of MC. In this context, ellagic acid has preventive properties for AD and it can be used to prevent cholinergic dysfunctions and oxidative stress associated with Alzheimer’s-type dementia [29]. In a noteworthy study, anti-dementia effects of ellagic acid and its glucoside conjugate thru AChE inhibition in glutamate-treated SH-SY5Y human neuroblastoma cells have been experimentally reported [30]. In another hydride study, it has been reported biosafety and anti-ChE activity of ellagic acid [31]. This phytocompound was the strongest ChE binder among all phytocompounds of MC in the present study and its modifications may decrease its toxicity to be considered an anti-AD drug-like compound. Esculin or aesculin belong to the coumarins with useful pharmaceutical applications. For instance, esculin improves cognitive weakness in diabetic nephropathy [32] as well as the anti-ChE activities of esculin and its congeners have been reported previously (e.g., [33]). This phytocompound also was in second-order as an AChE binder, while its toxicity is not tolerable to be classified as an anti-AD lead-like molecule and its quantitative structure and activity relationship analysis may offer us less toxic ChE binders in future computational and experimental efforts. The ChE inhibitory effect of an essential oil containing *alpha*-cadinol (5%) has been reported [34]. Estragole is known as p-allylanisole and methyl chavicol is a phenylpropene with possible carcinogenic or genotoxic effects. Our findings showed that estragole is a weak binder of ChE with high toxicity. In vitro AChE inhibitory properties of carvacrol and its derivatives have been reported [35, 36]. This study cautiously supported the dual anti-AD or antiinsectant potentials of carvacrol as a moderate ChE binder. Patuletin is a chromone that belongs to flavonols that possess potential immunosuppressive and anti-arthritic activity [37]. Patuletin has been categorized as top specific AChE binder in the present study. A spathulenol-rich extract derived from aerial parts of *Anthriscus nemorosa* (M. Bieb.) Spreng. (Apiaceae) has been reported as a strong AChE inhibitor [38]. The ChE inhibitory effects of terpinolene and its congeners as therapeutic agents in AD and as an antiinsectant have been reported previously (e.g., [39, 40]).

According to the findings of ADMET and toxicity, benzyl valerate, bicyclic monoterpenoids (isobornyl acetate and myrtenyl acetate), monoterpene (p-cymol), and cyclic monoterpene (terpinolene) would be lead-like or even drug-like candidates with putative anti-AD activity, while the rest compounds reported here, would be considered as putative antiinsectants (vide supra). However, the bioinformatics prediction was conducted to investigate the putative interactions of myrtle phytocompounds against target proteins involved in the pathogenesis of AD (e.g., [41–43]). Finally, future investigations are requested to dig deeper into the polypharmacology of formulations and compounds of myrtle to treat AD or be employed as antiinsectants.

## Conclusion

Methyl alcohol extract of berries of MC showed overt anti-ChE activity against AChE and BChE. While the leaves of the plant itself showed less inhibitory activity on both enzymes. The in silico results were comparable with experimental findings and an array of phytochemicals found in MC including ellagic acid, esculin, patuletin, *beta*-chamigrene, *alpha*-bulnesene, *beta*-bourbonene and (Z)-*gamma*-bisabolene had more negative BA with AChE, while ellagic acid, semimyrtucommulone, esculin, myrtucommulone A, patuletin and Juniper camphor in MC had more negative BA BChE. All compounds reported in MC showed acceptable ADMET while they were weak inhibitors of HERG therefore may lead to cardiotoxicity in large doses. This study proposed two categories of phytocompounds, namely, low class (Class I) toxic substances including beta-bourbonene, isobornyl acetate, benzyl valerate, myrtenyl acetate, *p*-Cymol, and terpinolene which can be used as phyto-nootropics (anti-AD) for symptomatic therapy of AD or high class (class III) toxic substances including ellagic acid, esculin, alpha-bulnesene, myrtenyl acetate, myrtucommulone A and B, and spathulenol which can be used as antiinsectants. In sum, the most specific and strongest AChE binders derived from MC was beta-bourbonene which was marketed as a flavoring agent with permeation to the brain, however its intestinal absorption should be improved to be considered as a candidate for AD. The patuletin and *alpha*-cardinal can be considered antiinsectants, and further experimental investigations are requested to assess their biopharmaceutical effects. This study categorized the pristine or natural phytocompounds of myrtle mostly as antiinsectants due to their toxic classes, however structural amendment and stereoselective synthesis like adding sulphonate or sulphamate groups to these phytocompounds may decline their toxicity and make them more suitable candidates for considering in preclinical investigations of AD.

### Supplementary Information

**Additional file 1.**

## Data Availability

The datasets of the current study are available from the corresponding author.

## Abbreviations

MC: *Myrtus communis* L.
AChE: Acetylcholinesterase
BChE: Butyrylcholinesterase
ChE: Cholinesterase
AD: Alzheimer’s disease
DTNB: 5,5´-Dithiobis [2-Nitrobenzoic Acid]
PDB: Protein Data Bank
BA: Binding affinity
ADMET: Absorption, distribution, metabolism, excretion and toxicity
BBB: Blood-brain barrier
GIT: Human gastrointestinal tract
HERG: Human ether-a-go-go-related gene
RO5: Lipinski’s rule of five
OH: Oxyanion hole
AS: Anionic subsite
AP: Acyl pocket
PAS: Peripheral anionic site
